# H_2_S Sensors: Synthesis, Optical Properties, and Selected Biomedical Applications under Visible and NIR Light

**DOI:** 10.3390/molecules28031295

**Published:** 2023-01-29

**Authors:** Dongning Liu, Winston Hessler, Maged Henary

**Affiliations:** 1 Department of Chemistry, Georgia State University, Atlanta, GA 30303, USA; 2 Center of Diagnostics and Therapeutics, Georgia State University, Atlanta, GA 30303, USA

**Keywords:** H_2_S sensor, cyanine dye synthesis, UV/Vis spectroscopy, ICT, TICT, FRET, PET, azide reduction

## Abstract

Hydrogen sulfide (H_2_S) is an essential signaling gas within the cell, and its endogenous levels are correlated with various health diseases such as Alzheimer’s disease, diabetes, Down’s syndrome, and cardiovascular disease. Because it plays such diverse biological functions, being able to detect H_2_S quickly and accurately in vivo is an area of heightened scientific interest. Using probes that fluoresce in the near-infrared (NIR) region is an effective and convenient method of detecting H_2_S. This approach allows for compounds of high sensitivity and selectivity to be developed while minimizing cytotoxicity. Herein, we report a review on the synthesis, mechanisms, optical properties, and selected biomedical applications of H_2_S sensors.

## 1. Introduction

Hydrogen sulfide (H_2_S) is the third most important endogenous signal gas that cells use [1,2], after carbon monoxide and nitric oxide. It mediates multiple physiological and pathological processes [3,4]. While crucial to normal biological function, H_2_S can prove an extremely toxic gas at higher concentrations. Some basic physicochemical and thermodynamic properties of H_2_S are listed in Table 1 [5].

A simple molecule, H_2_S is a marker of some of society’s most prevalent maladies. Cardiovascular disease, diabetes, and Down’s syndrome have all been associated with an increased level of H_2_S [6]. It is known to regulate myriad biological functions in vivo; oxidative stress, insulin signaling, and cell growth and death are all mediated by this species [7,8]. Energy available to the cell can also be affected by levels of H_2_S because it is involved in the inhibition of adenosine triphosphate (ATP) [9,10,11,12,13].

**Scheme 1:**
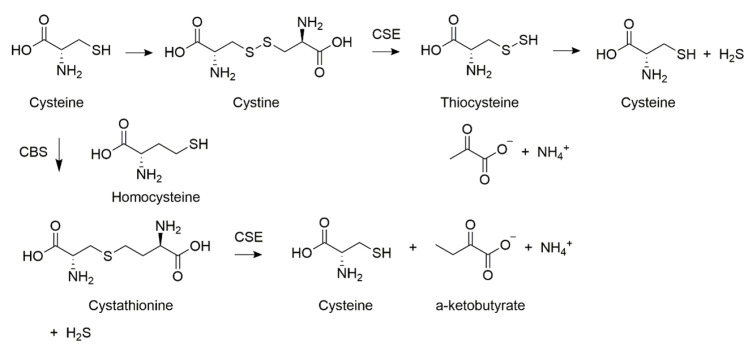
Endogenous hydrogen sulfide synthesizing pathways. Reprinted with permission from Ref. [15]. Copyright ©2010 American Chemical Society.

Biosynthesis of H_2_S in mammals is carried out by the enzymes cystathionine β-synthase (CBS), 3-mercaptopyruvate sulfotransferase (3-MST), and cystathionine γ-lyase (CSE) [14]. The endogenous pathways are laid out in Scheme 1 [15]. Homocysteine acts as the substrate for CBS, with H_2_S and cystathionine being liberated. The second mode involves a “desulfurization” reaction, whereby sulfur is cleaved from cysteine directly without its oxidation. Furthermore, it has been reported that the activities of these two enzymes in different cells and tissues are correlated to several health disorders [16,17,18]. For these reasons, H_2_S has become an important medium in the detection of disease [19,20,21].

Traditional methods of H_2_S detection, such as electrochemical analysis and gas chromatography, require a sample of living cells from target tissues [22,23]. However, since H_2_S can exist at levels as low as 30 μM in human blood, such methods are inadequate for detecting the analyte in the blood [24]. With these limitations in mind, the discovery of highly sensitive and selective detection methods for H_2_S is of peak interest [25,26,27,28,29,30]. Traditional methods also present the inherent drawbacks of trauma from conducting biopsies, inconsistent concentrations of the analyte within the target tissue, and an overall more tedious process. To improve results, various probes have been synthesized for the selective and specific detection of H_2_S [31,32,33].

Recent studies have shown the visible and NIR probes to be favorable due to their high-yield synthesis, modular structures, low cytotoxicity, and high sensitivity and selectivity [34,35,36,37,38,39,40,41,42,43,44,45,46]. Importantly, they work in real time and require nothing but an injection [47]. In the past few years, much work has been put into shortening response times, reducing Stokes shifts, and increasing the fluorescence wavelengths of these compounds [48,49,50,51]. Such findings would provide healthcare workers with new tools to prevent, detect, and treat various diseases.

Most probes featured in this review comprise cyanines and coumarins, operate via the mechanisms of nucleophilic addition and azide group reduction, and produce signals in the NIR region. Their synthesis, optical properties, and applications in the biomedical field are the main focus of this paper. In addition, we discuss the attributes unique to these sensors and challenges in their synthesis while speculating on the future direction of H_2_S probe development [52,53,54,55,56].

## 2. Different Mechanisms of H_2_S Detection

There are many kinds of H_2_S probes which have been designed and synthesized to date [57,58,59]. In the subsequent sections, we summarize two different mechanisms of H_2_S detection reported over the last 7 years [60,61,62,63,64,65,66,67,68,69,70,71,72,73,74,75,76,77,78,79,80]. These include probes (Table 2) based on the principles of nucleophilic attack [60,61,62,63,64,65,66,67,68,69,70,71,72,73,74] and reduction of azide groups [75,76,77,78,79,80]. Probes of each type can detect the H_2_S concentration at different biological levels. For each approach, we can provide examples detailing the structure and synthesis of the pertinent probes. Then, the optical properties and physical characteristics are laid out. Lastly, biomedical applications of these H_2_S probes are discussed.

### 2.1. Nucleophilic Addition to the Conjugated System for H_2_S Detection

Under the physiological condition of pH 7.6, H_2_S was deprotonated to HS^−^. This species was then able to be added to the conjugated system by way of nucleophilic attack onto the electrophilic center of the probe. In this way, cyanine dyes were able to detect H_2_S. Such a mechanism has four different modes of signal transduction. These include intramolecular charge transfer (ICT) [61,62,63], twisted intramolecular charge transfer [64,65,66] (TICT), fluorescence resonance energy transfer (FRET) [67,68,69], and photoinduced electron transfer (PET) [72,73,74]. Each of these processes involves the transfer of energy within the probe in the presence of the analyte. These mechanisms are discussed individually under the banner of H_2_S detection by way of nucleophilic addition to a conjugated system.

#### 2.1.1. Intramolecular Charge Transfer (ICT)

This is the process of converting light energy into chemical energy. An example is photosynthesis, where plants use sunlight to carry out key charge separation processes in the production of food. The ICT process depends on the relationship between intensities of the donor and acceptor in the system. The donor resides at the HOMO energy level, and the acceptor resides at the LUMO. Moreover, the energy gap between the two can serve to insulate or conduct depending on the circumstances, which determines the electronic coupling degree between the two orbitals [61,62,63].

##### Synthesis and Mechanism of H_2_S Probes Based on ICT

**Scheme 2:**
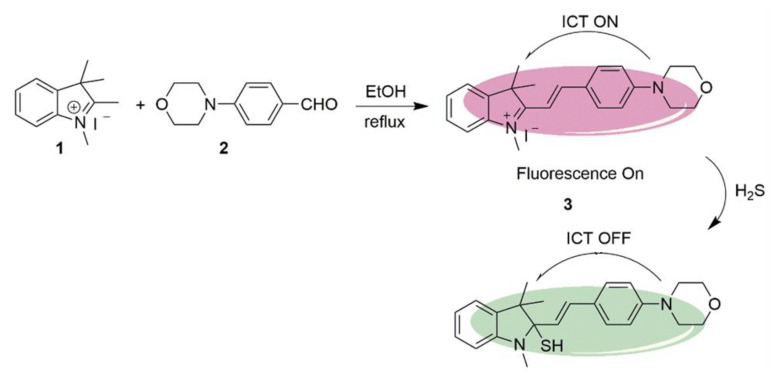
Synthetic route and detection mechanism of H_2_S probe **3**.

An example of a H_2_S probe performing on the basis of ICT is the morpholinostyryl probe **3**, as illustrated in Scheme 2. It was synthesized by Li et al. following the reaction of indolium salt **1** with aldehyde **2**. Under reflux in ethanol, a yield of 70% was achieved. The probe behaves as a switch, with fluorescence turning on and off in the absence and presence of H_2_S, respectively. This occurs because H_2_S, behaving as the nucleophile, attacks the indolium moiety to break conjugation. The result was a loss of fluorescence in the presence of the H_2_S at levels as low as 30 μM [61].

**Scheme 3:**
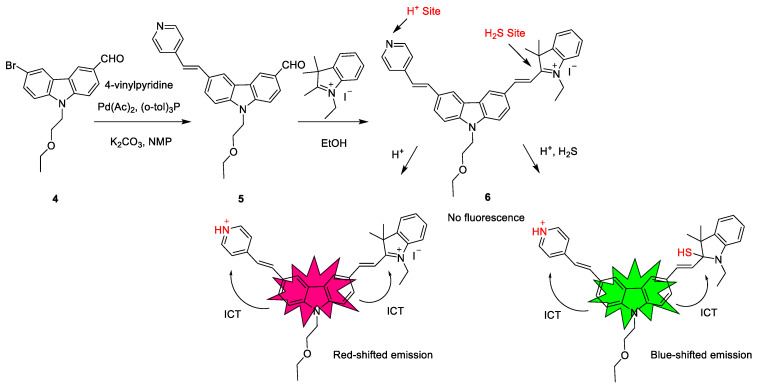
Synthetic route and detection mechanism of probe **6**.

In the previous example of a H_2_S probe, the detection is indicated by a loss of fluorescence. A different probe was synthesized by Yong Liu et al. to have a hypsochromic shift upon binding with the H_2_S analyte. Scheme 3 shows the production of probe **6** following a two-step reaction. First, the bromine of compound **4** is substituted with 4-vinylpyridine under basic conditions using a Pd(Ac)_2_ catalyst to yield compound **5**. Next, the conjugated system is extended following a Vilsmeier–Haack coupling with an indolium salt. The resulting probe **6** contains two separate sites where the fluorescence wavelength can be altered. Protonation can occur at the pyridine moiety to result in a bathochromic shift, and the indolium moiety can undergo nucleophilic attack in the presence of H_2_S to give a hypsochromic shift. As it is able to distinguish between H_2_S present in acidic versus nonacidic conditions, probe **6** can be useful in detecting the H_2_S analyte in different parts of the body. This is possible because the fluorescence wavelength can easily be measured by spectroscopy, and whichever shift is observed can indicate the environment in which the analyte was present. For instance, tumors are known to be more acidic than the surrounding healthy tissue while blood is normally slightly alkaline [62].

**Scheme 4:**
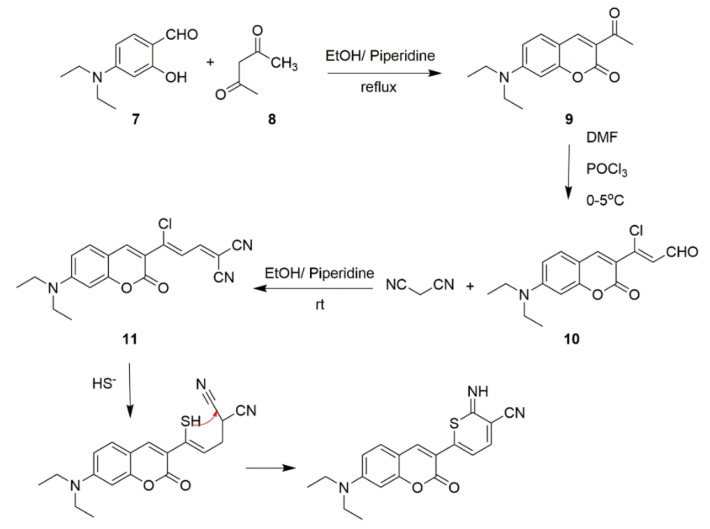
Synthetic route and detection mechanism of H_2_S probe **11**.

A novel on/off NIR fluorescent probe was developed by Fengzao Chen et al. which is unique in that it can detect H_2_S simultaneously by cyclization to form the six-member ring. Its synthesis and ICT mechanism are laid out in Scheme 4, involving three steps before being able to bind to H_2_S. The aldehyde derivative **7** and the 1,3-dicarbonyl compound **8** are refluxed under basic conditions to yield coumarin derivative **9**. The resulting ketone group of **9** is formylated via a Vilsmeier–Haack coupling reaction with DMF in the presence of POCl_3_ to yield compound **10**. Subsequent condensation with malononitrile produced compound **11** at a yield of 60%. This step is carried out at room temperature using EtOH and piperidine as solvents, and it results in the conjugated system being lengthened, as well as the addition of the strongly electron-donating cyano groups. This final product is then able to detect H_2_S under physiological conditions. The deprotonated species HS^−^ substitutes the chloride to give a thiol which then undergoes a proton transfer with one of the cyano groups. A six-membered ring is formed, and this can be detected by the shift in fluorescence wavelength [63].

##### Optical Properties of ICT H_2_S Probes

**Figure 1:**
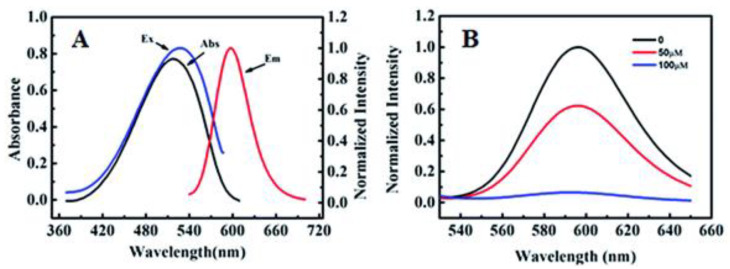
(**A**) Absorption and fluorescence spectra of probe **3**. (**B**) Fluorescence spectra of probe **3** (10 μM) in H_2_S at varying concentrations. Reprinted with permission from Ref. [61]. Copyright ©2017 Royal Society of Chemistry.

Referring to the on/off H_2_S probe synthesized by Li et al. in Scheme 2, probe **3** was shown to absorb light at a wavelength of 520 nm and fluoresce at 596 nm, as shown in Figure 1A. Its fluorescence intensity was then measured at 600 nm in the presence of H_2_S at varying concentrations. Figure 1B reveals that the signal of probe **3** was strongest with the analyte absent, but its signal was unobservable at a concentration of 100 μM of H_2_S. These results prove that probe **3** is a useful H_2_S sensor [61].

**Figure 2:**
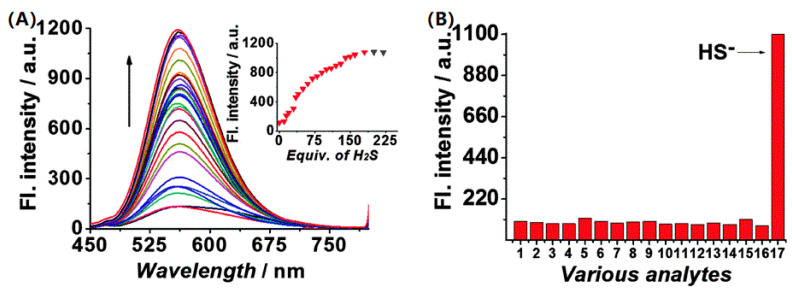
(**A**) Fluorescence spectra of probe **6** (10 μM) in pH 4.4 PBS buffer solution (containing 5% DMSO) with the addition of H_2_S. (**B**) Fluorescence responses of probe **6** at 405 nm in the presence of: 1. Probe 6; 2, Cl^−^; 3, K^+^; 4, Ca^2+^; 5, Mg^2+^; 6, GSH; 7, H_2_O_2_; 8, Hcy; 9, HSO_3_^−^; 10, I^−^; 11; Cys; 12, NO_2_^−^; 13, S_2_O_3_^2−^; 14, SO_3_^2−^; 15, SO_4_^2−^; 16, Zn^2+^; 17, HS^−^ [62]. Reprinted with permission from Ref. [62]. Copyright ©2016 Royal Society of Chemistry.

H_2_S probe **6** reported by Yong Liu et al. (Scheme 3), which displayed a bathochromic shift upon detection of the H_2_S analyte, was also found to exhibit spectral changes at varying pH of H_2_S. The absorption and fluorescence spectra of probe **6** at different pH values are shown in Figure 2A,B, respectively. Furthermore, probe **6** is highly sensitive to H_2_S under acidic conditions (Figure 2A). As presented in Figure 2B, probe **6** has good selectivity of H_2_S over various other sulfur-containing molecules such as SO_4_^2−^, S_2_O_3_^2−^, and cysteine [62].

**Figure 3:**
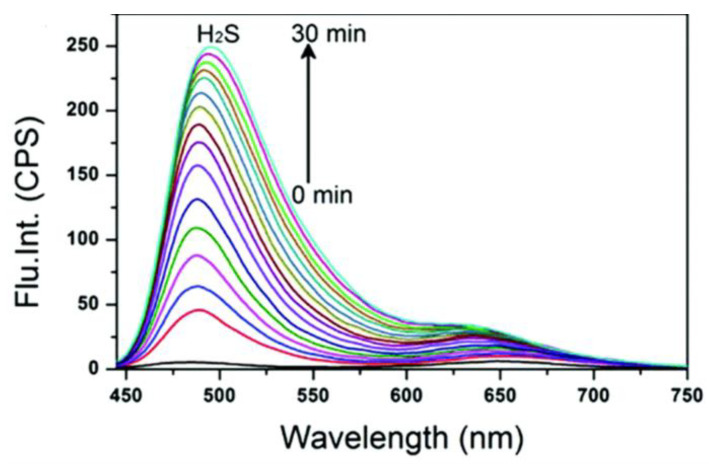
Time-dependent fluorescence spectral changes of probe **11**. Reprinted with permission from Ref. [63]. Copyright ©2018 Royal Society of Chemistry.

The third H_2_S probe operating on the ICT principle, probe **11** (Scheme 4), was also studied to undergo changes to its fluorescence spectra at 490 nm by Fengzao Chen et al., as shown in Figure 3. Intensity of fluorescence took 30 min to reach a plateau in the presence of H_2_S, at which point there was only marginal increase in signal strength with time [63].

##### The Application of H_2_S Probes Based on ICT

**Figure 4:**
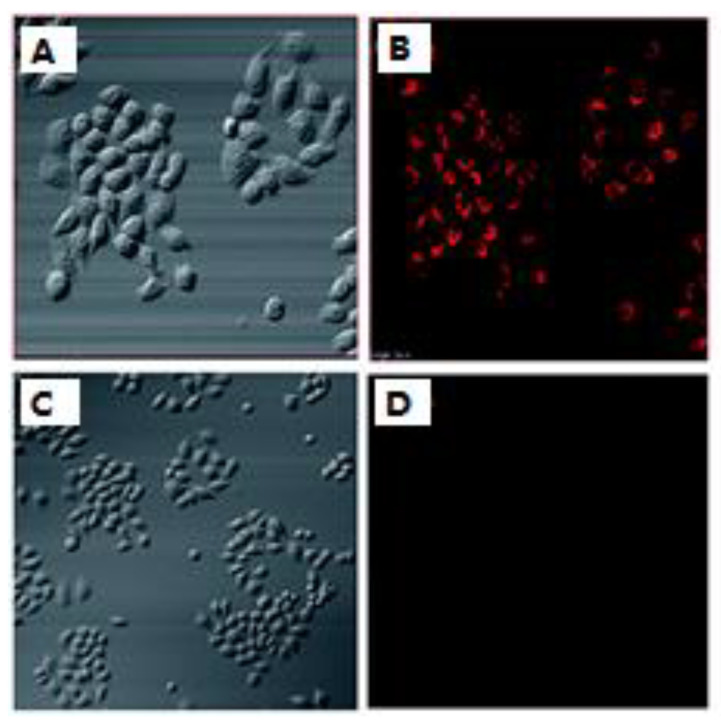
(**A**) Bright-field images of cells treated with probe **3**. (**B**) Bright-field images of cells preincubated with probe **3** and NaHS. (**B**,**D**) are fluorescence images of (**A**,**C**), respectively. Reprinted with permission from Ref. [61]. Copyright ©2017 Royal Society of Chemistry.

The immortalized cells of Henrietta Lacks, who died of advanced cancer in 1951, were used to further bolster the ability of probe **3** (Scheme 2) to detect H_2_S in living cells. This sensor penetrated the cell membrane as shown by the contrast between bright-field and fluorescence images of cells incubated with probe **3** (Figure 4A,B). Overlaying the bright-field and fluorescence images of cells treated with probe **3** verified this claim. Conversely, there was no visible fluorescence upon addition of H_2_S to the culture (Figure 4C,D). These images indicate that probe **3** could effectively penetrate the membrane and was selective toward H_2_S in living cells [61].

**Figure 5:**
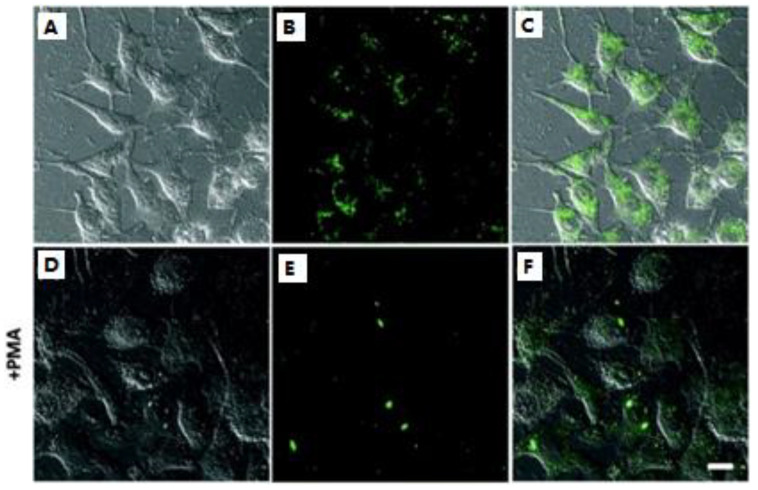
Fluorescence imaging of the endogenous H_2_S in the lysosomes: (**A**–**C**) the cells incubated with 5.0 μM probe **6** only; (**D**–**F**) the cells pretreated with PMA and then incubated with probe **6**. (**A**,**D**) bright-field images; (**B**,**E**) fluorescence; (**C**,**F**) merged. Reprinted with permission from Ref. [62]. Copyright ©2016 Royal Society of Chemistry.

Probe **6** (Scheme 3) was used to detect endogenous H_2_S in the lysosomes of A549 cells. To highlight the probe’s ability to detect H_2_S, an assay was performed both with and without the presence of PMS (12-O-tetradecanoylphorbol 13-acetate, 4β,9α,12β,13α,20-pentahydroxytiglia-1,6-dien-3-one 12-tetradecanoate 13-acetate), which was used to reduce endogenous H_2_S levels. Figure 5 illustrates that probe **6** did indeed detect the analyte within the lysosomes of the A549 cells (A–C), but the signal was reduced upon the addition of the H_2_S inhibitor, PMA (D–F). The addition of the inhibitor serves to prove the probe’s selectivity toward H_2_S and not simply any sulfur-containing analyte. The study confirms the ability of probe **6** to detect endogenous H_2_S in the cell lysosome under the green channel [62].

**Figure 6:**
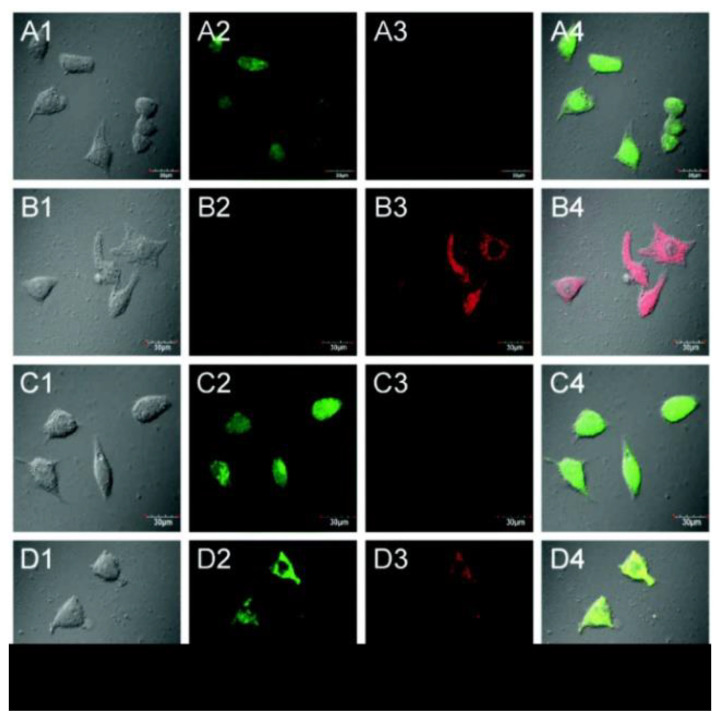
(**A**) Cells incubated with only probe **11**; (**B**) cells were preincubated with NEM and subsequently treated with probe **11**; (**C**) cells pretreated with NEM followed by treatment with probe **11** and GSH; (**D**) cells pretreated with 0.6 mM NEM followed by probe **11** and then with H_2_S. Column 1, bright field; column **2,** green channel; column **3,** red channel; column **4,** merged images of columns **1**, **2**, and **3**. Reprinted with permission from Ref. [63]. Copyright ©2018 Royal Society of Chemistry.

Probe **11** (Scheme 4) was used to detect H_2_S levels in MCF-7 cells. As shown in Figure 6, a strong fluorescence signal was observed in the green channel from 470–530 nm with H_2_S present (Figure 6(D2)), but hardly any was seen in the red channel from 620–680 nm (Figure 6(D3)). Upon addition of probe **11** following treatment with the H_2_S inhibitor NEM, only red-channel signals were seen (Figure 6(B4)). This means that, without H_2_S, probe **11** would show red channel signals. In the groups with supplemental H_2_S in addition to the inhibitor, the phenomenon was quite different; all others were only detectable in the green channel (Figure 6C,D). H_2_S causes a blue shift, and the signal is shown in the green channel. These results proved that probe **11** has use as an effective tool in detecting H_2_S in MCF-7 cells [63].

#### 2.1.2. Twisted Intramolecular Charge Transfer (TICT)

This is a process of electron transduction which is always linked by a single bond between donor and acceptor units. The twist of a single bond is an essential component of this process, unlike the ICT, which sees a lateral translation of electrons across a rigid structure. The TICT process occurs upon absorption of an electron. The excited molecular structure becomes twisted and then relaxes to the ground state, emitting light at a longer wavelength. Many TICT-based fluorescent probes are potential detectors for some small biological molecules [64,65,66].

##### Synthesis and Mechanism of H_2_S Probes Based on TICT

**Scheme 5:**
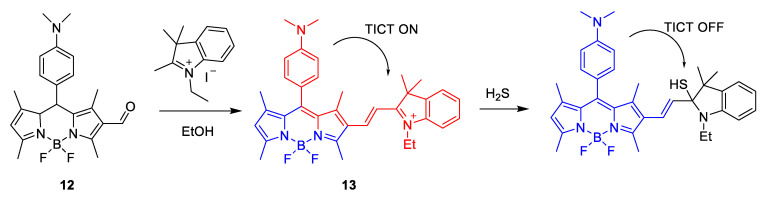
Synthetic route and detection mechanism of probe **13**.

Mingguang Ren et al. synthesized the first H_2_S TICT probe **13** (Scheme 5) based on the BODIPY scaffold. Probe **13** was obtained by the reaction between compound **12** and the indolium salt under reflux in EtOH. The salt was condensed with the aldehyde group in compound **12** to form probe **13** at 60% yield. For this probe, the combination of the conjugated structure of indolium with the BODIPY group turned on the TICT process, which showed a strong fluorescence. In the presence of H_2_S, the indolium moiety underwent a nucleophilic attack, thereby quenching the TICT process. Probe **13** could detect the H_2_S levels in living cells [64].

**Scheme 6:**
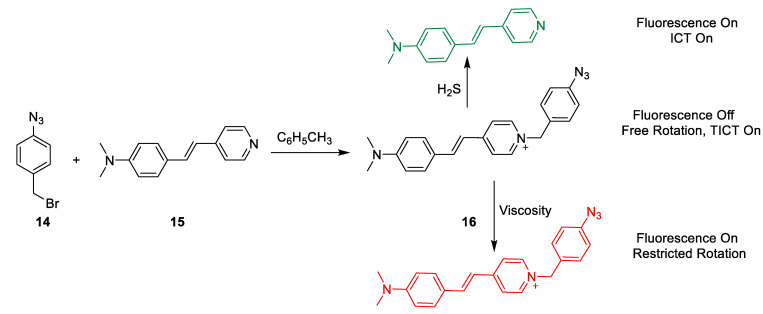
Synthetic route and detection mechanism of probe **16**.

Songjiao Li et al. designed H_2_S probe **16** to monitor levels of H_2_S in cells (Scheme 6). This compound performs according to the TICT mechanism in the NIR region of the EM spectrum. Under reflux conditions in toluene, the pyridine ring of compound **15** is alkylated by the azide derivative **14** to produce probe **16** at a yield of 90% In the absence of the H_2_S analyte, probe **16** does not show a fluorescence signal due to the free rotation in the TICT state. Upon the addition of H_2_S, the pyridine undergoes nucleophilic attack, and the azide moiety is liberated. At this point, fluorescence was induced. In this way, probe **16** could target the mitochondrion and visualize H_2_S at different concentrations in HeLa cells [65].

**Scheme 7:**
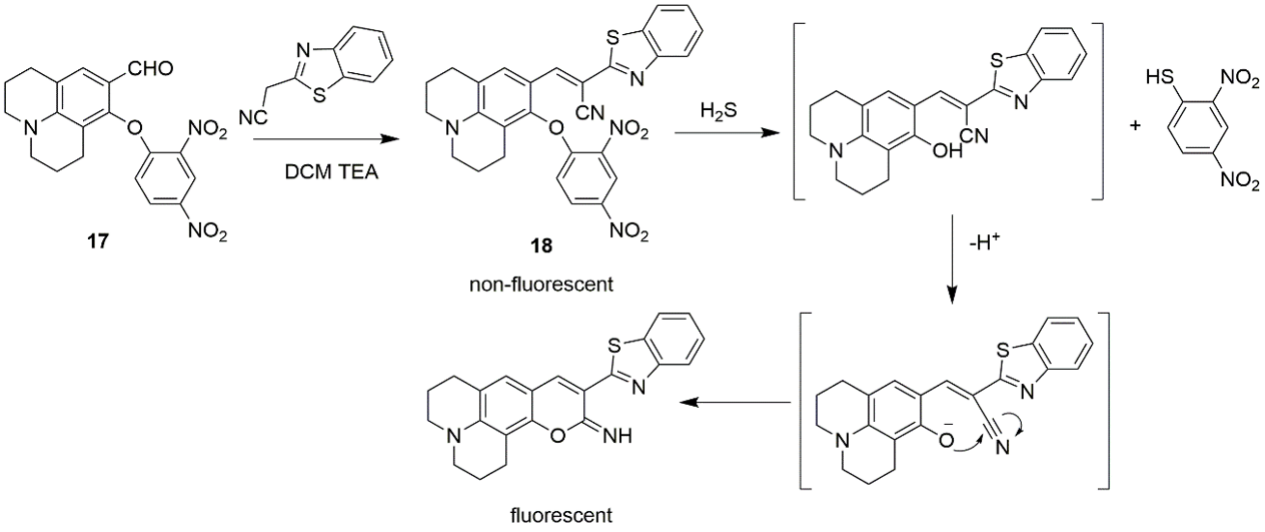
Synthetic route and detection mechanism of probe **18**.

Yongru Zhang et al. designed the dinitrophenyl H_2_S fluorescent probe **18**. Basic conditions facilitated the condensation of the benzothiazole derivative with the aldehyde of compound **17** to produce probe **18** in 12% yield. The mechanism via which H_2_S was sensed occurs via the analyte undergoing a nucleophilic aromatic substitution on the dinitrobenzene ring. In the process, 2,3-dinitrobenzothiol was liberated, and the cyano group of the intermediate was reduced to an imine (Scheme 7). The formation of a six-membered ring brought conjugation to the molecule, and this could be detected by spectroscopy. In this way, probe **18** could detect endogenous levels of H_2_S within living cells [66].

##### Optical Properties of H_2_S Probes Based on TICT

**Figure 7:**
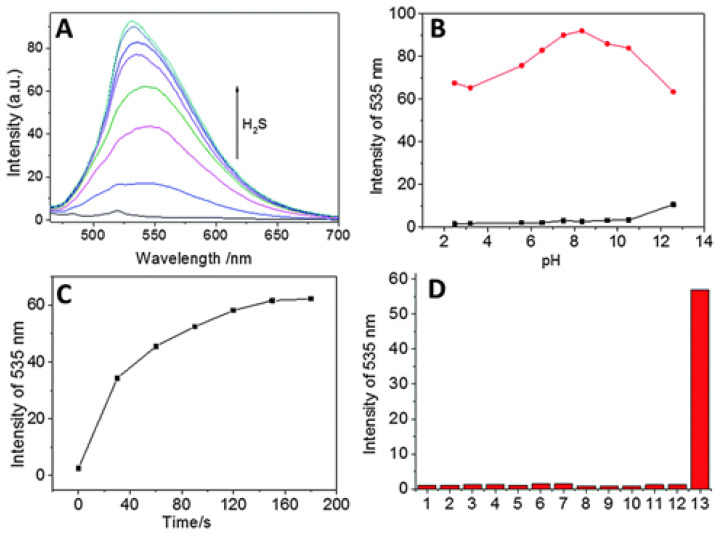
(**A**) Fluorescence spectra of probe **13** treated with Na_2_S (0–100 μM); (**B**) fluorescence intensity of probe **13** in the absence (black) and presence (red) of Na_2_S, and at varying pH; (**C**) time course of fluorescence intensity of probe **13** in the presence of Na_2_S; (**D**) fluorescence intensities of probe **13** treated with various relevant analytes (1) probe only; (2) F^−^; (3) GSH; (4) H_2_O_2_; (5) Hcy; (6) I^−^; (7) Cys; (8) NO_2_^−^; (9) S_2_O_3_^2−^; (10) SO_3_^2−^; (11) HSO_3_^2−^; (12) NO_3_^−^; (13) Na_2_S, in PBS buffer (pH 7.4, containing 50% DMF as a cosolvent). Reprinted with permission from Ref. [64]. Copyright ©2016 Royal Society of Chemistry.

Mingguang Ren et al. investigated the optical properties of probe **13** in the presence of H_2_S (Scheme 5). A detection plateau was observed at 100 μM (Figure 7A). The fluorescence intensity of probe **13** was shown to be stable across a wide pH range (Figure 7B). As shown in Figure 7C, it took up to 3 min for a maximum detection of fluorescence to be detected. Probe **13** was reported to have high specificity to H_2_S, as supported by Figure 7D; signals were negligible for a dozen other analytes, such as cysteine, SO_3_^2−^, and GSH. With these studies, it was proven that probe **13** has a high specificity, sensitivity, and selectivity toward H_2_S [64].

**Figure 8:**
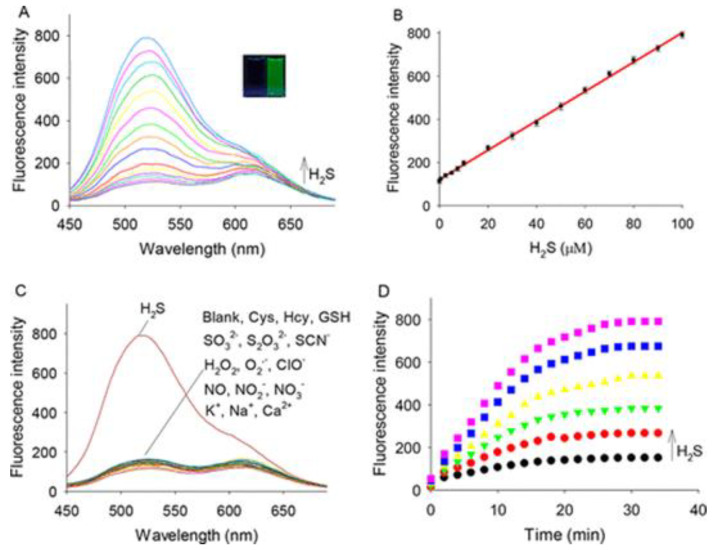
(**A**) Fluorescence spectra of probe **16**; (**B**) fluorescence intensity versus H_2_S concentrations; (**C**) fluorescence response of probe **16** toward H_2_S, GSH, and other interference species; (**D**) time-dependent fluorescence intensity change of probe **16** at varied concentrations of H_2_S. Reprinted with permission from Ref. [65]. Copyright ©2018 American Chemical Society.

Songjiao Li et al. investigated the fluorescence response of probe **16** (Scheme 6) to H_2_S in PBS (Figure 8A). As shown in Figure 8B, the limit of detection was as low as 0.17 μM. As shown in Figure 8C, with various interference chemicals including H_2_O_2_, NO_2_^−^, and Ca^2+^, there was no obvious fluorescence change of probe **16**, and, after 25 min, the fluorescence reached its maximum value (Figure 8D). These results suggest the probe **16** has high selectivity toward H_2_S [65].

**Figure 9:**
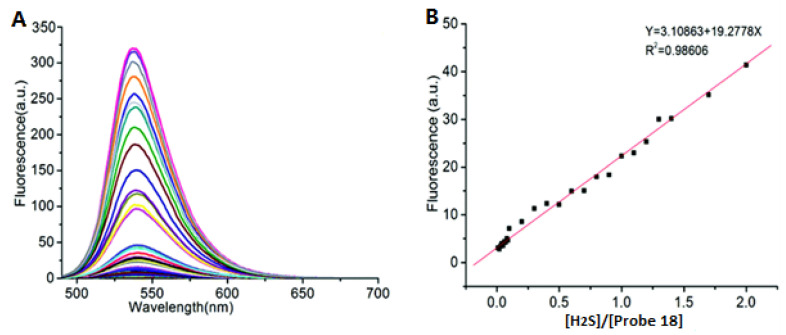
(**A**) Fluorescence spectra of probe **18** on the adjunction of Na_2_S in HEPES; (**B**) linear relationship between the fluorescence response at 537 nm and the concentration of Na_2_S in HEPES buffer. Reprinted with permission from Ref. [66]. Copyright ©2019 Royal Society of Chemistry.

Yongru Zhang et al. examined the sensitivity of probe **18** (Scheme 7) toward H_2_S. Na_2_S was used for the source of H_2_S. As outlined in Figure 9A, in HEPES buffer, probe **18** responded to Na_2_S with different fluorescence signals at 537 nm, which indicated an excellent linear relationship (R^2^ = 0.9860) at 537 nm. Moreover, a detection limit of 0.15 μM H_2_S was reported for probe **18** (Figure 9B). These results showed that probe **18** was a good monitoring system for H_2_S [66].

##### Applications of H_2_S Probes Based on TICT

**Figure 10:**
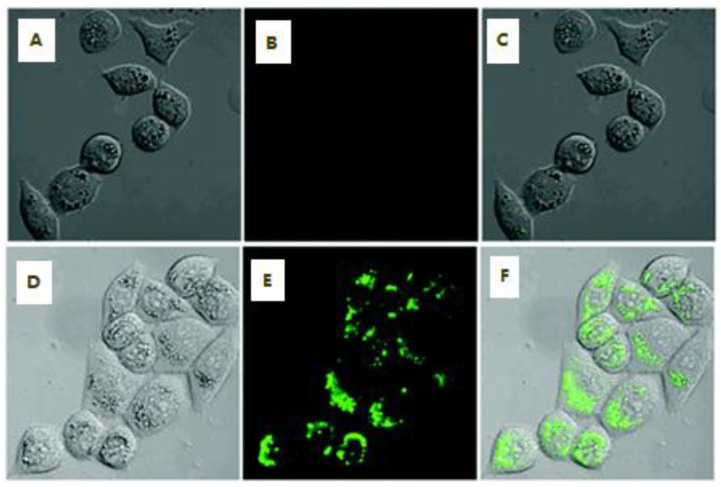
Visualization of H_2_S within HeLa cells; (**A**) HeLa cells and probe **13**, bright field; (**B**) cells and probe **13**, fluorescence; (**C**) merged image of (**A**,**B**); (**D**) cells, probe **13**, and Na_2_S, bright field; (**E**) cells, probe **13**, and Na_2_S, fluorescence; (**F**) merged image of (**D**,**E**). Reprinted with permission from Ref. [64]. Copyright ©2016 Royal Society of Chemistry.

Probe **13** (Scheme 5) was used to detect H_2_S in HeLa cells. As shown in Figure 10B, a weak fluorescence signal was associated with the cells being only incubated with the probe. In contrast, after probe **18** was treated with Na_2_S for 30 min, the HeLa cells had strong fluorescence signals (Figure 10E). The overlaid image (Figure 10F) clearly showed the green fluorescence in the HeLa cells. These results prove the utility of probe **13** as a H_2_S sensor [64].

**Figure 11:**
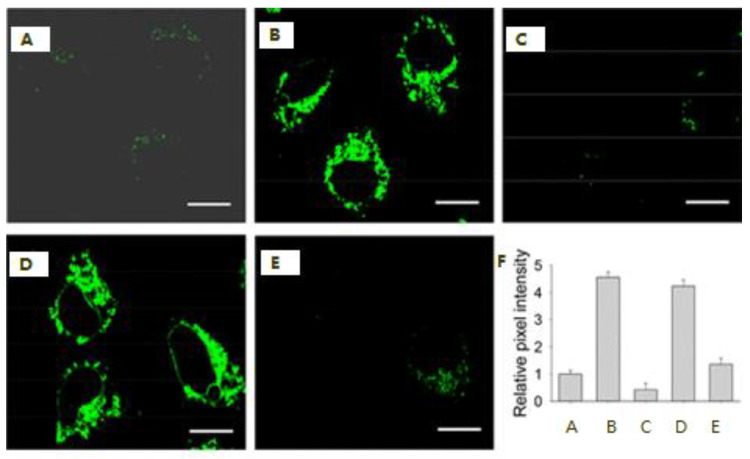
Fluorescence imaging of HeLa cells; (**A**) HeLa cells stained with probe **16**; (**B**) HeLa cells stained with probe **16** and then treated with Na_2_S; (**C**) HeLa cells preincubated with PAG and then treated with probe **16**; (**D**) HeLa cells stimulated with Cys and probe **16**; (**E**) HeLa cells stimulated with Cys, PAG, and probe **16**; (**F**) Intensities of (**A**–**E**). Reprinted with permission from Ref. [66]. Copyright ©2019 Royal Society of Chemistry.

Probe **16** (Scheme 6) was used to image H_2_S in HeLa cells, as shown in Figure 11. There was only a weak fluorescence signal when HeLa cells treated by the probe alone (Figure 11A). With the addition of Na_2_S to the cells, the green fluorescence signal increased (Figure 11B). However, when the H_2_S inhibitor PAG was added, the fluorescence signal was weak again (Figure 11C). This indicates that probe **16** could be useful in monitoring exogenous H_2_S in living cells. In contrast, the use of cystine, which could induce the production of endogenous H_2_S, leads to an increase in green signal once more (Figure 11D). When PAG and cystine were added concurrently, the fluorescence intensity in the HeLa cells was weak. This was due to the inhibition of H_2_S (Figure 11E,F) [66].

#### 2.1.3. Nucleophilic Addition Based on FRET

**Scheme 8:**
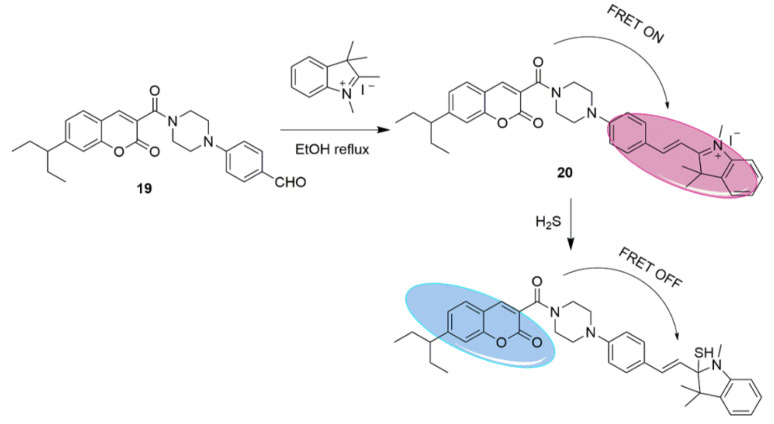
Synthetic route and detection mechanism of Probe **20**.

Fluorescence resonance energy transfer (FRET) occurs between an excited donor and an acceptor at the ground state. Donors emit in a shorter wavelength that overlaps with the acceptor’s absorbance. A nonradioactive energy transfer occurs, i.e., the FRET phenomenon, such that the fluorescence intensity of the donor is much lower than when it exists alone, while the fluorescence emitted by the acceptor is greatly enhanced. This energy transfer occurs without the appearance of a photon and results from long-range dipole–dipole interactions between the donor and acceptor. Fluorescence of excited species is quenched in the presence of the analyte (H_2_S), which transduces a signal [67,68,69]. As an example, in Scheme 8, the coumarin is excited and transfers energy to the indolium, which fluoresces. Upon H_2_S reacted with the indolium, the FRET process is interrupted, and the coumarin fluoresces.

##### Synthesis of H_2_S Probes Based on FRET

Xiao Feng et al. synthesized an NIR fluorescent probe operating according to the FRET principle. The coumarin derivative **20** was obtained via a single-step condensation reaction (Scheme 8). The aldehyde of compound **19** was allowed to react with the N-methyl indolium salt under reflux to form a new carbon–carbon bond. A good yield of 70% was reported, and the induced conjugation provided a means of detection through spectroscopy. During the detection of H_2_S, the indolium moiety undergoes nucleophilic attack. Conjugation was lost, thereby quenching the FRET process. It was noted that, under a single-wavelength excitation, this probe could show different signal changes with the H_2_S concentration [67].

**Scheme 9:**
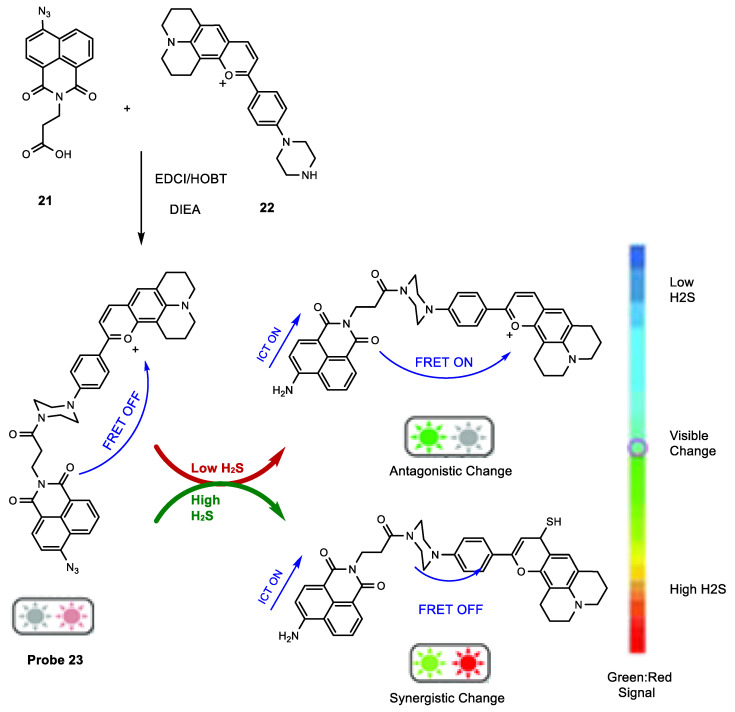
Synthetic route and detection mechanism of probe **23**.

Yunzhen Yang et al. designed a ratiometric fluorescent probe working via both the ICT and the FRET mechanisms. Scheme 9 illustrates the coupling reaction between the carboxylic acid group of **21** and the secondary cyclic amine of **22** with EDCI, HOBT, and DIEA as coupling conditions. The new carbon–carbon bond resulted in probe **23** with a yield of 70%. In response to H_2_S, probe **23** could exhibit different signals depending on the concentration of the analyte. At higher concentrations, the azide group was reduced to a terminal amine whose lone pair contributed to the conjugated system. The ICT was activated, and this was revealed by a signal with more red character. However, at lower concentrations, FRET was the indicator, with a signal of more blue character [68].

**Scheme 10:**
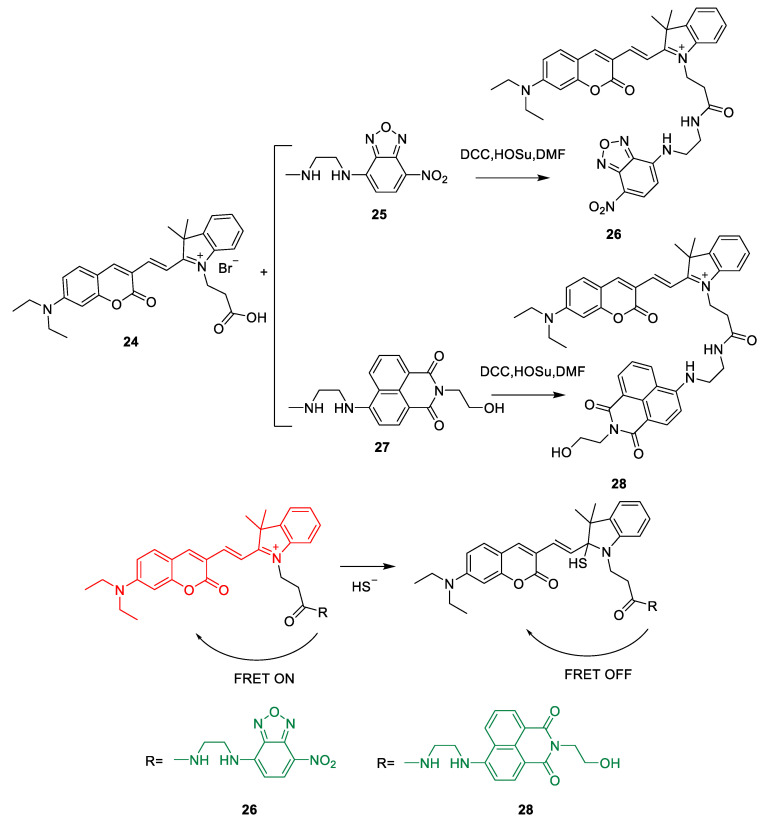
Synthetic route and detection mechanism of probe **26** and **28**.

Yuming Zhang et al. developed two ratiometric fluorescent H_2_S sensors. Both were formed following condensation reactions between the carboxylic acid group of the coumarin derivative merocyanine fluorophore **24** and the amine groups of the two green-emitting fluorophores **25** and **27**. Probes **26** and **28**, respectively, were obtained as shown in Scheme 10 using DCC chemistry and a HOSu catalyst. Low yields of 35% were reported. In detection of the H_2_S analyte, the indolium moieties of the probes underwent nucleophilic attack by H_2_S. FRET was turned off in the process, thereby transducing the signal [69].

##### Optical Properties of H_2_S Probes Based on FRET

**Figure 12:**
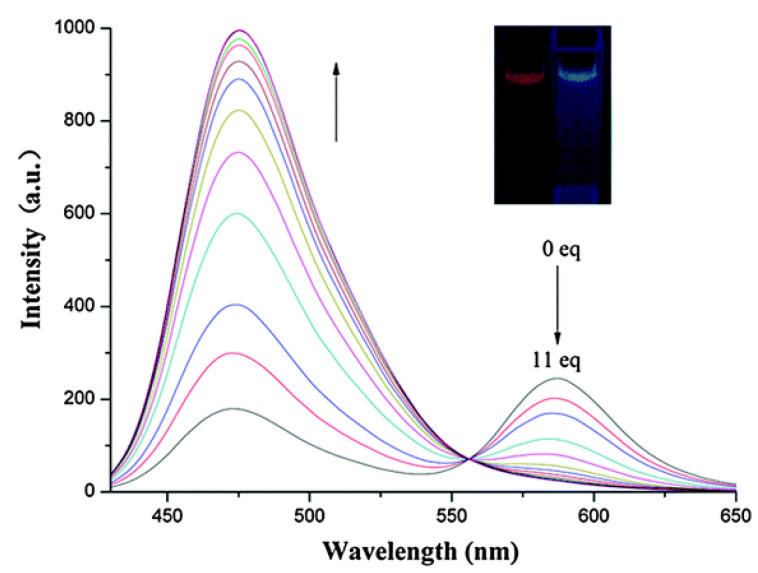
Fluorescence spectra of the probe **20** with the addition of Na_2_S in PBS buffer. Reprinted with permission from Ref. [67]. Copyright ©2016 Royal Society of Chemistry.

Xiao Feng et al. investigated the sensitivity of probe **20** (Scheme 8) for H_2_S. As shown in Figure 12, the fluorescence titration demonstrated that probe **20** had two distinct emission bands. Interestingly, as the concentration of H_2_S was increased, the ratio of these two peaks changed; the 587 nm signal became more intense while the 474 nm signal became less intense as H_2_S was added. The opposite was also found to be true; lower concentrations increased the lower wavelength signal and reduced the higher one. These findings prove that probe **20** could be potentially used to quantitatively determine the concentration of H_2_S in a sample [67].

**Figure 13:**
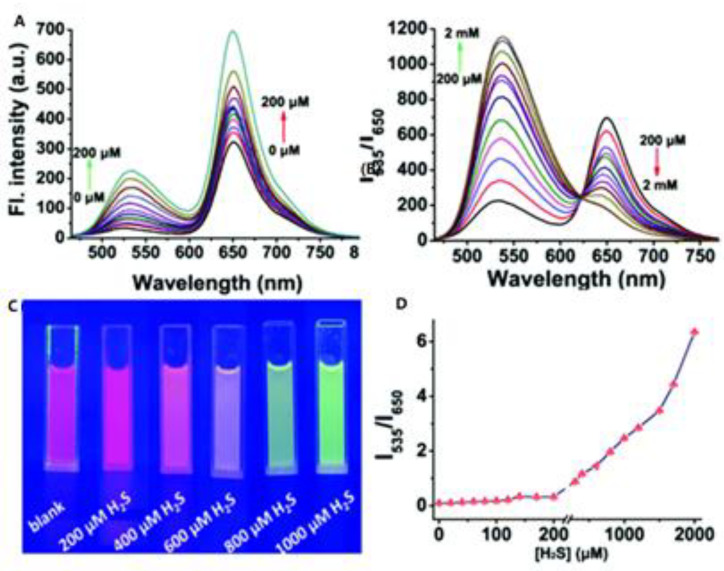
(**A**) Fluorescence spectra of probe 23 with NaHS in phosphate buffer; (**B**) fluorescence spectra of probe 23 with NaHS in phosphate buffer; (**C**) color change of the chemosensor 23 solution; (**D**) ratio values of fluorescence intensity of probe 23 with NaHS. Reprinted with permission from Ref. [68]. Copyright ©2019 Royal Society of Chemistry.

Yunzhen Yang et al. studied the optical properties of probe **23** (Scheme 9). The free probe was shown to emit at 650 nm (Figure 13A); however, with increasing concentrations of H_2_S, this peak at 650 nm decreased in favor of the emerging peak at 540 nm (Figure 13B). At concentrations below 200 uM, 650 nm was the major peak, whereas 550 nm was the major peak between a concentration of 200 uM and 2 mM. This phenomenon revealed an isosbestic point in Figure 13B at 620 nm. The color of emission varied depending on the concentration of H_2_S as illustrated in Figure 13C; more blue character was correlated to higher concentrations of the analyte. As shown in Figure 13D, concentrations of H_2_S below 200 μm gave peak ratios overwhelmingly favoring the peak at 650 nm. This agreed with the rest of the data because lower concentrations resulted in signals with redder intensity [68].

**Figure 14:**
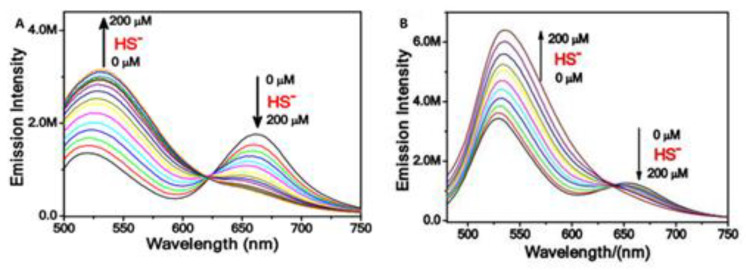
Emission spectra of probe **26** 10 μM (**A**)and **28** (**B**) in PBS solution in the presence of different amounts of HS^−^ excited at 475 nm. Reprinted with permission from Ref. [69]. Copyright ©2020 Royal Society of Chemistry.

Yuming Zhang et al. studied the optical properties of probe **26** (Scheme 10) and its ability to sense H_2_S. As shown in Figure 14A, when HS^−^ was added, the signal at 530 nm increased significantly, while the signal at 665 nm showed a decrease. Probe **28** was shown to behave similarly in Figure 14B. The isosbestic point resulted around 625 nm (Figure 14A) and 640 nm (Figure 14B). These results prove that both probes **26** and **28** could potentially serve as radiometric sensing probes for H_2_S.

##### The Application of H_2_S Based on FRET Probes

**Figure 15:**
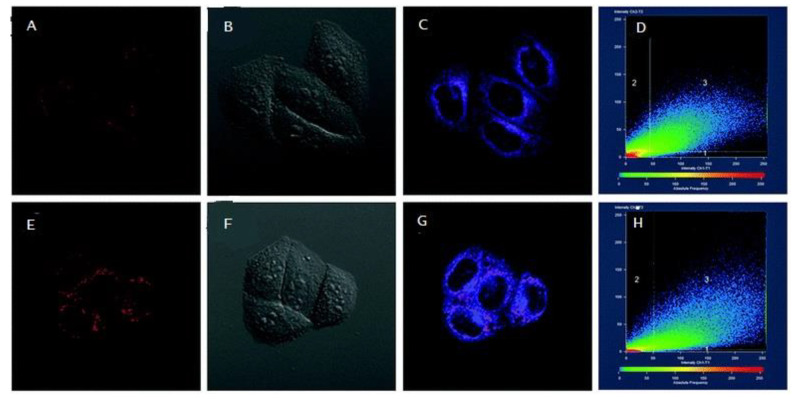
(**A**–**D**) Probe **20** with Mito Tracker Deep Red; (**E**–**H**) HeLa cells treated with Na_2_S for 1 h, followed by probe 20 for 1 h, and then incubated with Mito Tracker for 0.5 h; (**A**,**E**) HeLa cells with probe **20**, bright field; (**B**,**F**) MTDR; (**C**,**G**) merged image; (**D**,**H**) colocalization studies. Reprinted with permission from Ref. [67]. Copyright ©2016 Royal Society of Chemistry.

Probe **20**, the first mentioned sensor operating by FRET, was used to detect H_2_S in HeLa cells (Scheme 8). Figure 15 shows the probe colocalization in the cells both with (Figure 15E–H) and without (Figure 15A–D) the H_2_S analyte. When H_2_S was added, probe **20** was found to preferentially distribute in the mitochondrion, where it remained even after sensing for 1 h. This quality is displayed by the merged images of the cells under bright-field and deep-red wavelengths (Figure 15C,G). Furthermore, the probe’s photostability was evaluated by the HeLa cell images which were exposed for different lengths of time. Coefficients for quantitation of colocalization with the cells were reported as 0.924 and 0.891 (Figure 15D,H). These images suggest that probe **20** has use as a ratiometric tracker of H_2_S levels within the mitochondrion [67].

**Figure 16:**
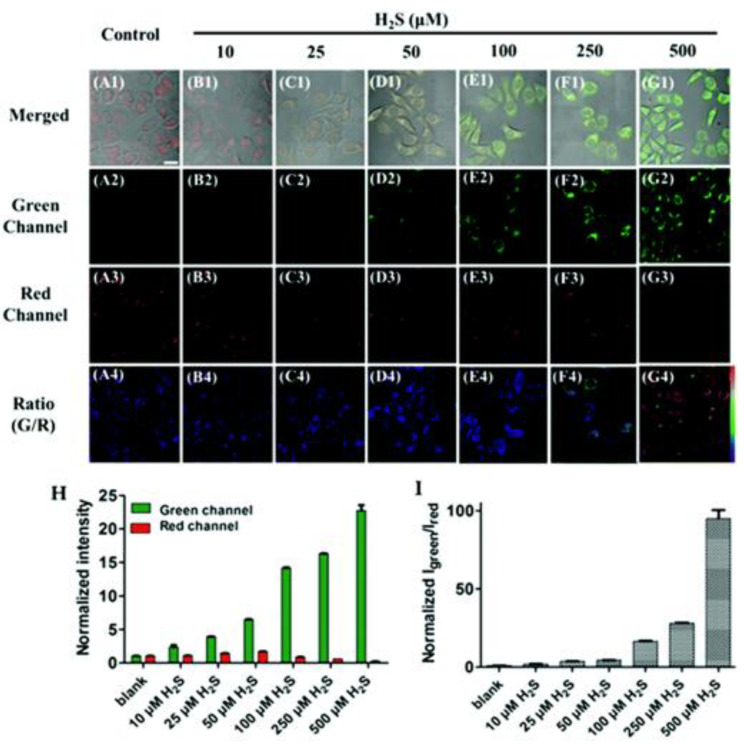
(**A**) Confocal fluorescence images of the chemosensor probe **23** responding to exogenous H_2_S in living cells. HeLa cells incubated with the chemosensor probe **23**, followed by treatment with NaHS; (**H**) normalized average fluorescence intensity of the green and red channels in (**A**–**G**); (**I**) normalized ratio of the fluorescence signals from the green channel and red channel in the panels (**A4**–**G4**). Reprinted with permission from Ref. [68]. Copyright ©2019 Royal Society of Chemistry.

Probe **23** (Scheme 9) was also used to detect H_2_S in the HeLa cells; however, unlike probe **20**, this compound was able to distinguish between high and low levels of concentration, with a cutoff at 50 μM (Figure 16I). In the control group, only weak fluorescence was shown (Figure 16A). As shown in Figure 16B–D, the intensities of fluorescence signals increased in both the green and the red channels at concentrations from 10 to 50 µM. As the concentration of NaHS was increased from 100 to 500 μM, the emission in the green channel maintained this enhancement (Figure 16(E2,F2,G2)); meanwhile, the emission in the red channel was weakened conspicuously (Figure 16(E3,F3,G3)). The ratios between the two signals were marked by a significant increase (Figure 16E–G). As shown in Figure 16H, the normalized fluorescence intensity of the green and red channels first exhibited a synergistic change (increased together) and then an antagonistic change (green channel increased while red channel decreased). Moreover, as shown in Figure 16I, the ratio of the signals first exhibited a slow increase; then, in the high range of the H_2_S concentrations, it increased significantly, which could help in identifying the cutoff point [68].

**Figure 17:**
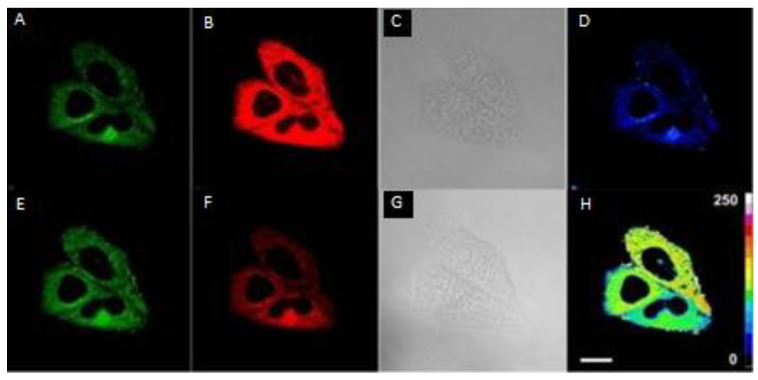
Confocal fluorescent microscopic images of probe **26** stained HepG-2 cells in the absence (**A**–**D**) and presence (**E**–**H**) of NaHS; (**A**,**E**) green channel; (**B**,**F**) red channel; (**C**,**G**) bright-field images; (**D,H**) pseudo-color ratio images obtained from green channel and red channel. Reprinted with permission from Ref. [69]. Copyright ©2020 Royal Society of Chemistry.

The third FRET-based H_2_S probe, **26**, was used to detect intracellular levels of the H_2_S analyte (Scheme 10). In Figure 17A, the green channel shows medium-intensity fluorescence while Figure 17B shows the red channel with strong emission. The combinatory image of Figure 17D indicates low intracellular H_2_S level in HepG-2 cells. When the cells were treated with NaHS for 30 min, the green channel was increased (Figure 17E), while the red channel was significantly reduced (Figure 17F), resulting in enhancement of the ratio image (Figure 17H). These results showed that probe **26** could detect the intracellular H_2_S [69].

#### 2.1.4. Photoinduced Electron Transfer (PET)

In PET fluorescent molecular probes, there is a photoinduced electron transfer between the fluorophore and the acceptor unit, which has a very strong quenching effect on fluorescence; usually, electrons are transferred from the donor to the excited fluorophore. Therefore, before binding to the analyte, the probe does not emit fluorescence or the fluorescence is very weak. Once the receptor is bound to the analyte, the photoinduced electron transfer is inhibited, which causes the fluorophore to emit fluorescence [72,73,74].

##### Synthesis and Mechanism of H_2_S Probes Based on PET

**Scheme 11:**
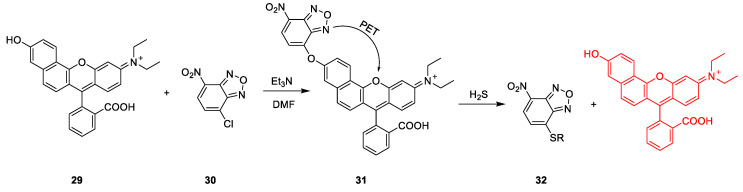
Synthetic route and detection mechanism of probe **31**.

Hanchuang Zhu et al. designed probe **31** according to the PET mechanism (Scheme 11). The chloro substituent of compound **30** was displaced under basic conditions, and its adjacent carbon was attacked by the hydroxy group of compound **29**. The resulting probe **31** served as the H_2_S sensor, and it achieved a yield of 70%. In the presence of H_2_S, nucleophilic attack occurred at the C–O bond para to the nitro group. As a result, probe **29** was formed again along with a thiol derivative of compound **32**. The PET was turned off in the process, and the H_2_S analyte was detected [72].

**Scheme 12:**
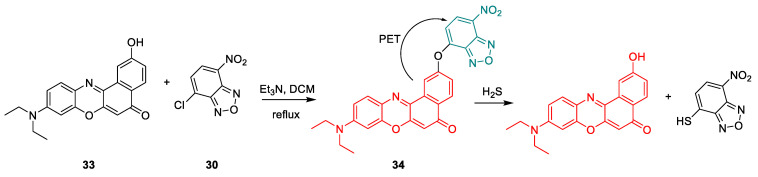
Synthetic route and detection mechanism of probe **34**.

Similar to the previous example, Jinshuai Lan et al. also produced an NIR probe capable of detecting H_2_S by PET (Scheme 12). Probe **34** was obtained through a coupling reaction between the chloro substituent of compound **30** and the hydroxyl group of compound **33**. This reaction produced probe **34** with a yield of 50%. In the presence of the H_2_S analyte, the NBD C–O bond was cleaved as indicated in Scheme 12, and the PET process was turned off [73].

**Scheme 13:**
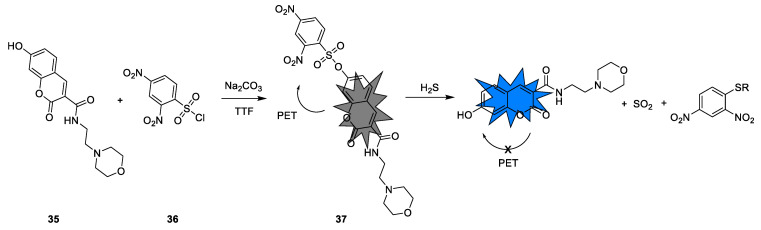
Synthetic route and detection mechanism of probe **37**.

Xinying Jing et al. developed a novel H_2_S probe **37** based on PET mechanism. Using a different approach from the previous two examples, the hydroxyl group of compound **35** reacted with the sulfonyl chloride group of compound **36** under basic conditions (Scheme 13). This coupling reaction produced compound **37** with a yield of 40%. The C–O bond of probe **37** underwent a nucleophilic attack by the H_2_S analyte, resulting in its cleavage. A species identical to staring material **35** and a thiol derivative of **36** were left as products. This reduction in conjugation quenches the PET, allowing for a signal to be detected [74].

##### Optical Properties of H_2_S Based on PET Probes

**Figure 18:**
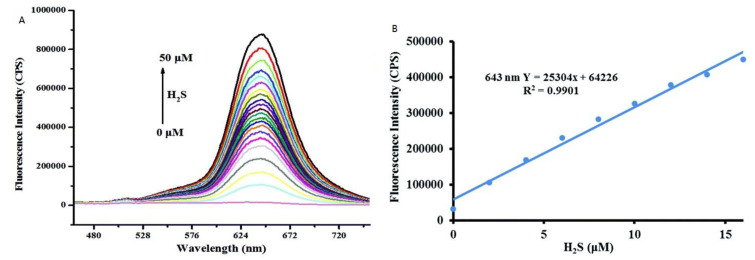
(**A**) Concentration-dependent fluorescence of probe **31** in the presence of H_2_S; (**B**) linear relationship between fluorescence intensities and biothiols. Reprinted with permission from Ref. [72]. Copyright ©2019 Royal Society of Chemistry.

The optical properties of H_2_S probe **31** (Scheme 11) were summarized by Hanchuang Zhu et al. as shown in Figure 18. Compound **31** displayed a fluorescence wavelength at 643 nm upon addition of H_2_S up to 50 μM (Figure 18A), demonstrating a high sensitivity toward the analyte. The definite linear relationship between the concentration of H_2_S and fluorescence intensity indicated that H_2_S could be detected accurately in levels as low as 6.2 × 10^−8^ mol·L^−1^ (Figure 18B). These results indicate that probe **31** has potential as a H_2_S sensor and deserves further research [72].

**Figure 19:**
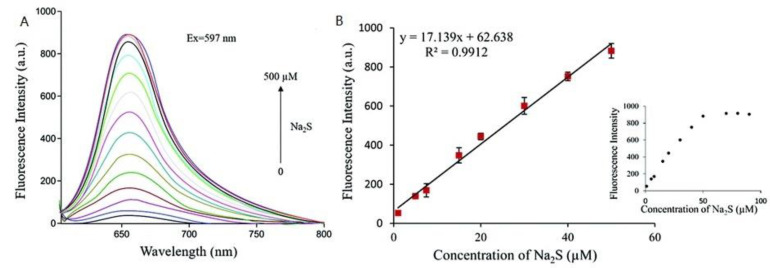
Fluorescence spectra changes of the probe **34** upon addition of increasing amount of Na_2_S. Reprinted with permission from Ref. [73]. Copyright ©2019 Royal Society of Chemistry.

Jinshuai Lan et al. presented the optical properties of probe **34** (Scheme 12; Figure 19A). The fluorescence signal at 650 nm increased as the concentration of Na_2_S increased from 0 to 500 μm. As such, a distinct linear relationship was reported between the concentration of the H_2_S analyte and the fluorescence intensity. The best fit line was used to report that S^2−^ concentrations under 60 μm could be accurately detected by probe **34** (Figure 19B) [73].

**Figure 20:**
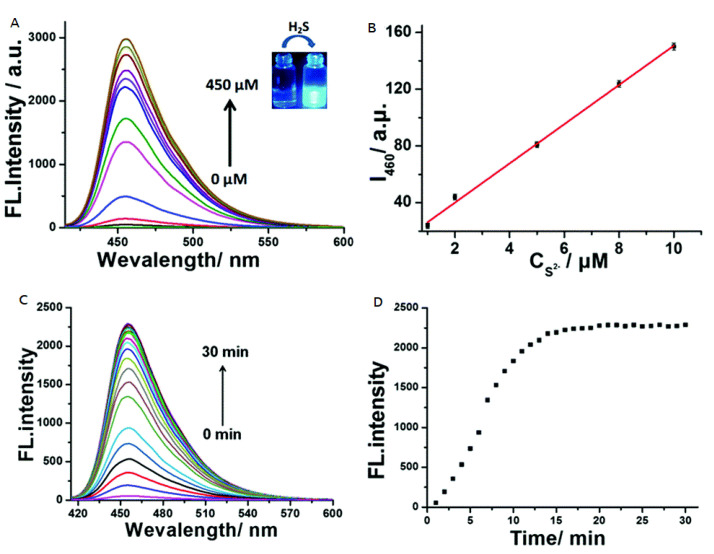
(**A**) Fluorescence spectra of the probe 37 in aqueous buffer; (**B**) linear relationship between the concentration of H_2_S and the fluorescence intensity; (**C**) fluorescence spectral changes of probe 37 with H_2_S; (**D**) Time–fluorescence intensity curve of probe 37 in the presence of H_2_S in buffer. Reprinted with permission from Ref. [74]. Copyright ©2019 Royal Society of Chemistry.

The optical properties of probe **37** in response to H_2_S (Scheme 13) were presented by Xinying Jing et al. in Figure 20A. Although the free probe did fluoresce weakly at 460 nm, this signal increased significantly upon addition of the H_2_S analyte. The strong linear relationship between the concentration of S^2−^ and fluorescence intensity at 460 nm (Figure 20B) suggests that the limit of detection of the probe could be as low as 2.07 × 10^−7^ M. As shown in Figure 20C, D, the fluorescence signal approached its maximum intensity after 15 min [74].

##### Applications of H_2_S PET Probes

**Figure 21:**
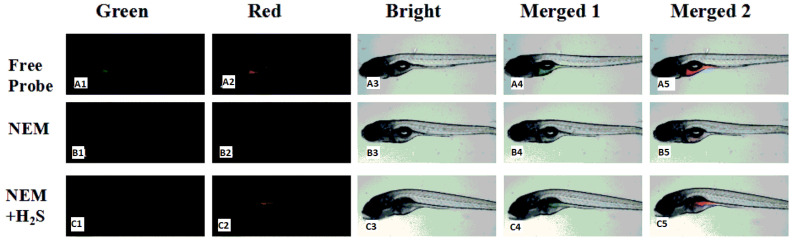
Zebrafish imaging: (**A**) zebrafish treated with probe **31**; (**B**) zebrafish preconditioned with NEM treated with probe **31**; (**C**) NEM-pretreated zebrafish treated with H_2_S then treated with probe **31**. Reprinted with permission from Ref. [72]. Copyright ©2019 Royal Society of Chemistry.

Zebrafish were used as living models to demonstrate the biological applications of probe **31** (Scheme 11). As shown in Figure 21A, when the zebrafish were only incubated with probe **31**, the images showed fluorescence in the green and red channels due to inherent biothiols within the cells. After treatment with the probe plus N-ethylmaleimide (NEM), a scavenger of biothiols, the zebrafish showed no fluorescence signal in either the green or the red channel (Figure 21B). Lastly, H_2_S and probe **31** were added (Figure 21C), which resulted in red and green fluorescence signals, as seen in Figure 21A. This strongly supports the potential use of probe **31** to detect both intracellular and extracellular H_2_S in zebrafish. Future research might discover applications of this compounds in other animal models [72].

**Figure 22:**
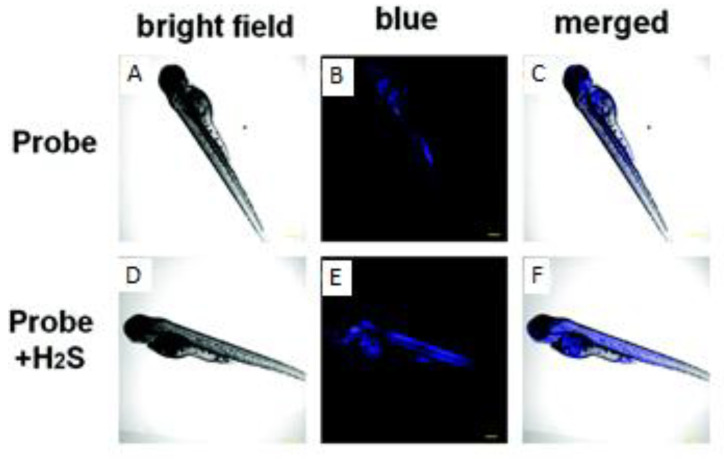
Fluorescence imaging of H_2_S by the probe 37 in zebrafish; (**A**–**C**) zebrafish were treated with the probe 37 solely; (**D**–**F**) zebrafish were treated with probe 37 and then incubated with H_2_S. (**A**,**D**) Bright-field images; (**B**,**E**) fluorescence images from the blue channel. Reprinted with permission from Ref. [74]. Copyright ©2019 Royal Society of Chemistry.

The biological applications of probe **37** (Scheme 13) were also demonstrated using zebrafish (Figure 22). Half an hour after being treated with only probe **37**, a fluorescence signal was seen, albeit dim, but this was solely due to intracellular biothiols (Figure 22B). Upon the addition of Na_2_S at 100 μM, the blue fluorescence was enhanced significantly by 20 min (Figure 22E). This proves that probe **37** is useful in detecting H_2_S in a living organism [74].

### 2.2. H_2_S Probes Based on Azide Group Reduction

In addition to nucleophilic addition, the reduction of azide groups is also a useful method for detecting H_2_S [75,76,77,78,79,80]. Typically, this involves a unit of hydrogen sulfide attacking an azide and reducing it to an amine via a reduction reaction, as opposed to nucleophilic attack of the H_2_S analyte to quench the fluorescence reaction. In the process, the overall electron distribution of the probe is altered, which can lead to an increase in fluorescence under certain conditions. These probes have been applied for both in vivo and in vitro testing by chemists to target the lysosomes and mitochondria of cells [79,80].

#### 2.2.1. Synthesis and Detection Mechanism of H_2_S Probes Based on Azide Group Reduction

**Scheme 14:**
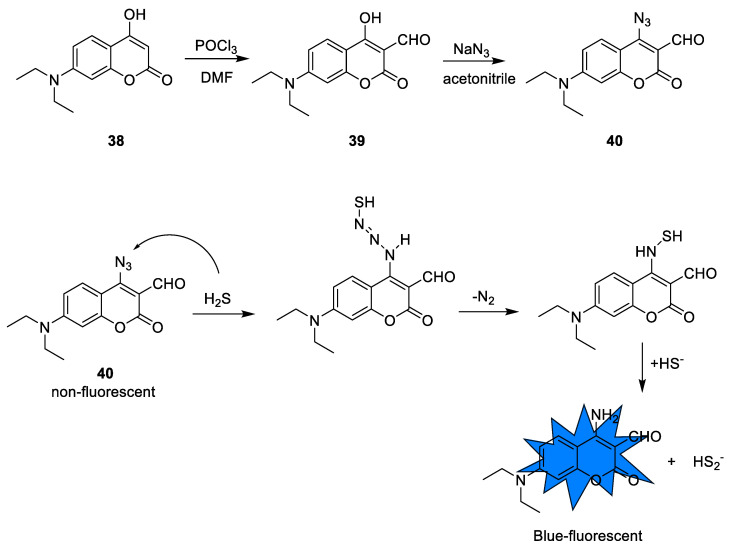
Synthetic route and detection mechanism of probe **40**.

One example of an NIR fluorescent H_2_S probe operating via azide reduction is compound **40** (Scheme 14), which was reported by Qian Fang et al. The synthesis of probe **40** is shown in Scheme 14. Vilsmeier–Haack formylation of coumarin derivative **38** using phosphoryl chloride produced compound **39**. Subsequent substitution of the hydroxyl group with sodium azide yielded probe **40** at 70%. In the presence of H_2_S, the azide underwent nucleophilic attack. The thioazide intermediate readily released a unit of nitrogen gas, and the resulting amine was protonated to make the molecule strongly fluorescent [75].

**Scheme 15:**
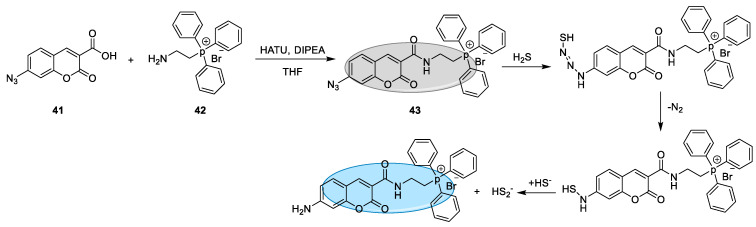
Synthetic route and detection mechanism of probe **43**.

Nithya Velusamy et al. reported a separate on/off probe, compound **43**, which could monitor H_2_S in living cells. With HATU and DiPEA in THF as coupling conditions, the carboxylic acid of compound **41** reacted with the terminal amine of compound **42** to form probe **43** at a yield of 40% (Scheme 15). In the presence of H_2_S, the azido group was reduced to an amino group, thereby transducing a signal in the blue region upon fluorescence [76].

**Scheme 16:**
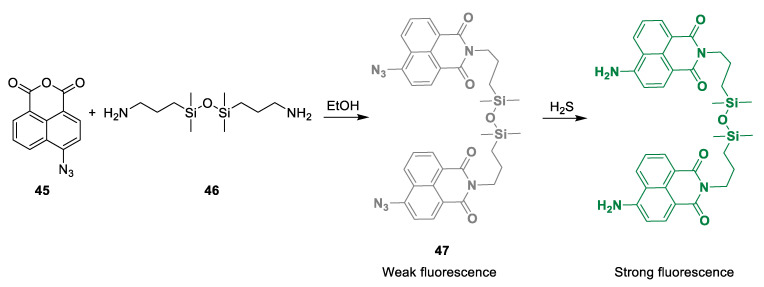
Synthetic route and detection mechanism of probe **47**.

A sort of H_2_S double probe was produced by Xiaoni Wang et al. Probe **47** comprised two probe moieties linked by a Si–O–Si bridge. Compounds **45** and **46** were condensed in EtOH to form compound **47** with a 70% yield. A strong green fluorescence signal was observed when the azido groups of probe **47** (Scheme 16) were attacked by H_2_S and reduced to amino groups [77].

#### 2.2.2. Optical Properties of Probes Based on Azide Group Reduction

**Figure 23:**
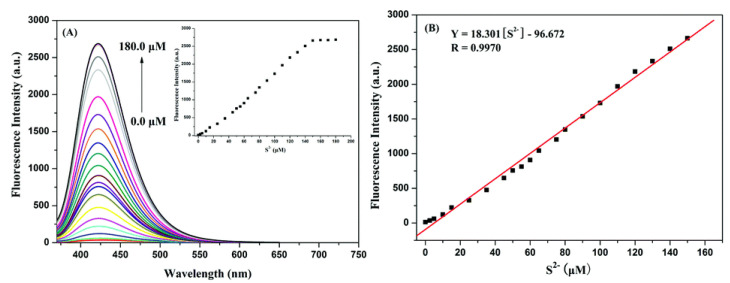
(**A**) Concentration-dependent fluorescence spectroscopy of probe **40** with Na_2_S; (**B**) linear correlation of (**A**). Reprinted with permission from Ref. [75]. Copyright ©2019 Royal Society of Chemistry.

The probe developed by Qian Fang et al. (Scheme 14) was investigated, and its optical properties were determined. Figure 23A shows that the fluorescence signal at 422 nm increased conspicuously while the concentration of H_2_S was raised to 180 μM. Manipulation of the fluorescence spectra provided a definite linear relationship between the fluorescence intensity and the concentration of Na_2_S [75].

**Figure 24:**
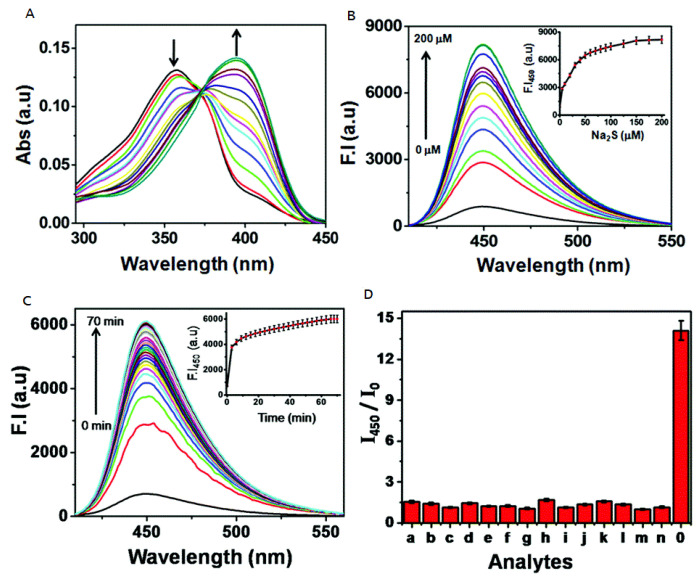
(**A**) UV/Vis absorption and (**B**) fluorescence spectra of probe **43**; (**C**) fluorescence change of probe **43** with Na_2_S; (**D**) fluorescence responses of probe **43** in the presence of various biologically important analytes: (a) cysteine (Cys), (b) H_2_O_2_, (c) NaNO_2_, (d) Cu(OAc)_2_, (e) Zn(OAc)_2_, (f) FeSO_4_, (g) FeCl_3_, (h) Na_2_CO_3_, (i) GSH, (j) NO, (k) Na_2_S_2_O_3_, (l) K_2_S_5_, and (m) N_2_S_4_. Reprinted with permission from Ref. [76]. Copyright ©2017 Royal Society of Chemistry.

Nithya Velusamy et al. reported the optical properties of probe **43** (Scheme 15; Figure 24). Absorption spectra showed a bathochromic shift as concentration of the H_2_S analyte was increased (Figure 24A), and fluorescence spectra indicated that the detection limit was as low as 10 μM (Figure 24B). As shown in Figure 24C, the fluorescence intensity reached a plateau after 70 min. Many sulfur-containing biomarkers were added to the probe, but only Na_2_S produced a strong signal (Figure 24D). These data show that probe **40** was very useful as a H_2_S probe because of its high selectivity and sensitivity [76].

**Figure 25:**
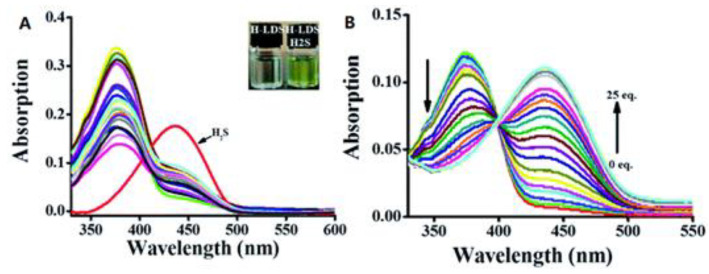
(**A**) Absorption spectra of probe 47 (10 μM) in the present of different ions (Cys, Glu, Gly, NaBr, NaCl, NaClO, NaCNS, NaF, BaCl_2_, CaCl_2_, CoCl_2_, CuCl_2_, FeCl_3_, MgCl_2_, NiSO_4_ ZnSO_4_, and SnCl_2_); (**B**) absorption spectra of probe 47 (10 μM) in aqueous solution upon addition of different amounts of H_2_S. Reprinted with permission from Ref. [77]. Copyright ©2020 Royal Society of Chemistry.

Figure 25 illustrates the optical properties of probe **47** (Scheme 16) as investigated by Xiaoni Wang et al. Figure 25A indicates a strong response to the addition of H_2_S and no response to other selected bioanalytes such as cysteine, glutamine, and glycine. The shift in absorption wavelength from 375 to 440 nm could even be detected by the naked eye. Increasing the concentration of H_2_S from 0 eq. to 25 eq. (equivalent volume of the probe), the absorption intensity of the peak at 375 nm gave way to another at 440 nm (Figure 25B). The isosbestic point shown in Figure 25B was around 400 nm. This selectivity shows that probe **47** has potential to be used as a H_2_S sensor [77].

#### 2.2.3. The Application of H_2_S Probe Based on Azide Group Reduction

**Figure 26:**
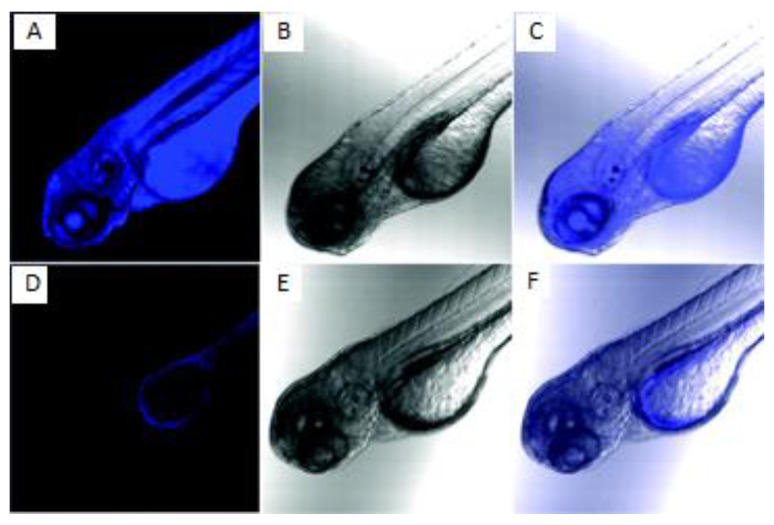
Fluorescence (left column), bright field (middle column) and merged (right column) images of 3-day old zebrafish. Top row: zebrafish incubated with probe 40 for 30 min and then treated with Na_2_S for 30 min. Bottom row: zebrafish incubated with probe 40 for 30 min. Reprinted with permission from Ref. [75]. Copyright ©2019 Royal Society of Chemistry.

Compound **40** (Scheme 14) was used to detect H_2_S in living zebrafish. Following the treatment with probe **40** only, weak fluorescence was seen. This was solely due to the H_2_S which would normally be found within cells (Figure 26D,F). However, after adding H_2_S and incubating with probe **40** for another 30 min, a strong blue fluorescence signal was observed (Figure 26A,C). These results show that probe **40** can be useful to detect H_2_S in living zebrafish [75].

**Figure 27:**
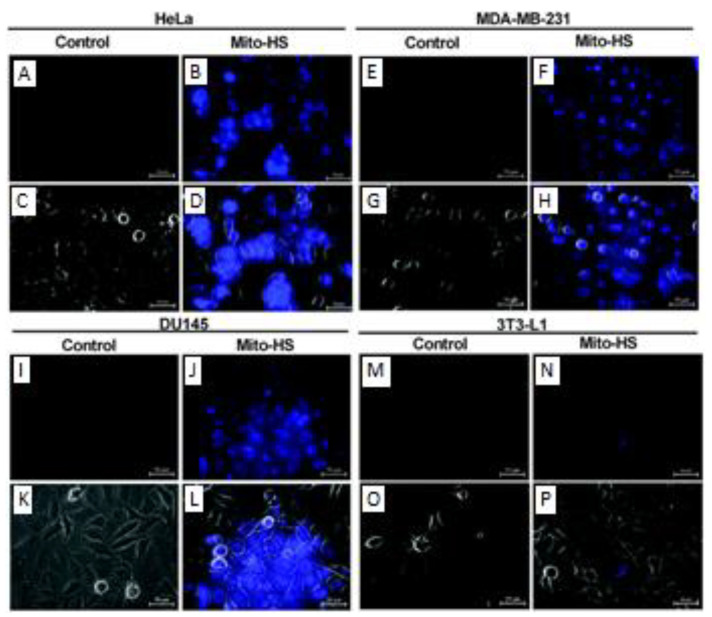
Fluorescence microscopy images of untreated HeLa (**A**), MDA-MB-231 (**E**), DU145 (**I**), and 3T3-L1 (**M**) cells and (**B**,**F**,**J**,**N**) the same cells, respectively, treated with probe 43 (5 μM). (**C**,**D**,**G**,**H**) and (**K**,**L**,**O**,**P**) are overlays of DIC bright-field images with the corresponding fluorescence images. Reprinted with permission from Ref. [76]. Copyright ©2017 Royal Society of Chemistry.

The presence of H_2_S was detected in four different cell lines by probe **43** (Scheme 15). HeLa (Figure 27A–D), MDA-MB-231 (E–H), DU145 (I–L), and 3T3-L1 cells (M–P) were all used to demonstrate this ability. As shown in all control groups, the fluorescence signals were all very weak without the probe present. This is because endogenous H_2_S levels are insufficient to elicit a signal. However, adding H_2_S increased the signal dramatically in most cases (Figure 27D,H,L). The 3T3-L1 cell line displayed the last pronounced response to H_2_S by probe **43** (Figure 27P). There is strong evidence that probe **43** is a useful probe for H_2_S in living cells [76].

**Figure 28:**
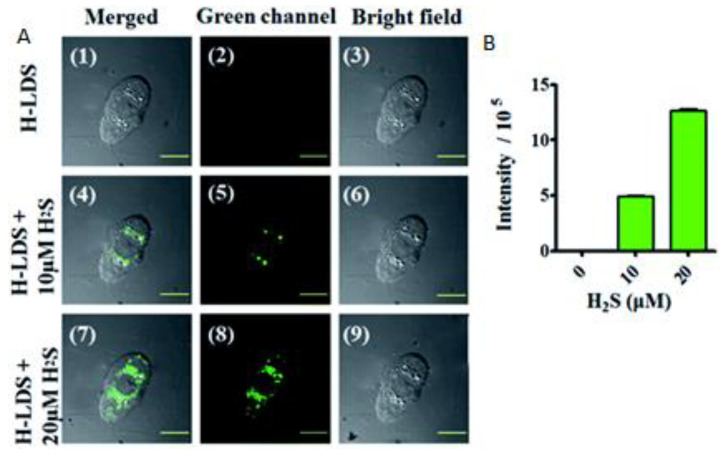
Confocal microscopy images of exogenous H_2_S in living cells using probe **47**; (**A**) (**1**–**3**) The fluorescence images of probe **47** in HeLa cells; (**4**–**6**) probe **47** treated with H_2_S; (**B**) analysis of the quantitative fluorescence intensity in (**A**) using Image software. Reprinted with permission from Ref. [77]. Copyright ©2020 Royal Society of Chemistry.

Another compound was investigated as a potential sensor for H_2_S in HeLa cells. Probe **47** (Scheme 16) displayed only weak fluorescence intensity within the cells by themselves (Figure 28(A1–3)). However, when the cells were treated with H_2_S, an obvious green fluorescence was seen (Figure 28(A4–9)). Varying concentrations of H_2_S were used, and their quantitative fluorescence intensities are plotted in Figure 28B. Together, this study shows that probe **47** has potential in detecting elevated levels of H_2_S within HeLa cells [77].

## 3. Summary and Outlook

Hydrogen sulfide is one of the most important gases in living organisms, and any abnormal changes in the concentration of it in vivo have been associated with many kinds of diseases [81]. Therefore, the development of highly sensitive H_2_S probes for use in living cells has become an important goal for research groups all around the world. Chemists have developed an assortment of on/off sensors which can be visualized with readily available NIR cameras. In this review, we exhibited many hydrogen sulfide probes based on several detection mechanisms, discussed their optical properties, and provided examples of their biological applications. In summary, most of the characteristic absorption peaks of fluorescent probes were in the visible and near-infrared range. The probes have exhibited promising, applicable prospects both in vivo and in vitro. These probes show excellent characteristics, such as good selectivity, low toxicity, and high sensitivity.

The diverse roles played by H_2_S in the body provide myriad avenues of scientific discovery. Already, it has been demonstrated that H_2_S is an important signaling gas in the nervous system [82]. For example, neurodegenerative diseases such as Parkinson’s disease have been linked to a reduction in its levels, and targeted supplementation of H_2_S to affected structures has proven to be therapeutical [81,83]. Not only certain diseases but also aging itself is connected to endogenous H_2_S levels because it has powerful properties against oxidative stress [84]. For this reason, future work should go into improving the applicability of these probes and perhaps integrating them with therapeutic techniques. The sensitivity of these probes needs to be improved to allow for more precise quantitative analysis, i.e., a better map of the H_2_S concentration gradient in the body. Additionally, by further extending the excitation wavelengths of the probes, signaling could improve due to less background interference. Lastly, applying H_2_S sensors to living models such as rodents would allow researchers to observe how these sensors behave under more variables. Perhaps one day, novel H_2_S-based treatments will be added to our pharmacopeia to slow, halt, or even reverse some of the most unavoidable afflictions we face.

## Figures and Tables

**Table 1:** Basic physicochemical and thermodynamic properties of H_2_S. Reprinted with permission from Ref. [5]. Copyright ©2018 American Chemical Society.

Properties	Data
Dipole moment	0.97 D
Solubility (in H_2_O)	110 mM/atm, 25 °C
	210 mM/atm, 0 °C
Boiling temperature	−60.2 °C
Density (25 °C, 1 atm)	1.36 kg/m^3^
IR	ν_1_ 2525, 2536 cm^−1^
	ν_2_ 1169, 1184, 1189 cm^−1^
	ν_3_ 2548 cm^−1^
^1^H-NMR	0.52 ppm
p*K*_1_	6.98
p*K*_2_	>17 at 25 °C
λ_max_ (HS^−^)	230 nm
ε	8 × 10^3^ M^−1^·cm^−1^
Henry’s law coefficient (298 K)	0.087135 mol solute/mol water atom
Detection threshold by human nose	0.02–0.03 ppm
Lethal dose	>500 ppm
Δ_f_*G*° (H_2_S)	−28 kJ/mol
Δ_f_*G*° (HS^−^)	+12 kJ/mol
Δ_f_*G*° (S^2−^)	+86 kJ/mol
*E*°′ (S^•−^, H^+^/HS^−^)	+0.91 V
*E*°′ (HS_2_^−^, H^+^/2HS^−^)	−0.23 V

**Table 2:** A summary of all probes discussed in this review.

Probe Number	Detection Mechanism	Fluorescence Emission	LOD	Bioapplication	Ref.
**3**	ICT	600 nm	15 nM	HeLa Cells	[61]
**6**	ICT	625 nm	3.2 μM	A549 cells and liver tissue	[62]
**11**	ICT	490 nm	75 nM	MCF-7 cells	[63]
**13**	TICT	535 nm	1.7 μM	HeLa Cells	[64]
**16**	TICT	510 nm	0.17 μM	HeLa cells	[65]
**18**	TICT	537 nm	0.15 μM	Ec1 cells	[66]
**20**	FRET	587 nm	40 nM	HeLa cells	[67]
**23**	FRET	650 nm	0.2 μM	HeLa cells and zebrafish	[68]
**26**	FRET	665 nm	0.5 μM	HepG-2 cells	[69]
**31**	PET	643 nm	62 nM	RAW 264.7 cells and zebrafish	[72]
**34**	PET	650 nm	40 nM	HepG2 and L02 cells	[73]
**37**	PET	460 nm	0.2 μM	HeLa cells and zebrafish	[74]
**40**	Azide reduction	422 nm	3.5 nM	HeLa cells and zebrafish	[75]
**43**	Azide reduction	450 nm	24.3 nM	HeLa cells	[76]
**47**	Azide reduction	550 nm	0.57 μM	HeLa cells	[77]
